# Rice (*Oryza sativa*) Bran and Soybean (*Glycine max*) Meal: Unconventional Supplements in the Mead Production

**DOI:** 10.17113/ftb.60.01.22.7183

**Published:** 2022-03

**Authors:** Geiza Suzart Araújo, Gislane Oliveira Ribeiro, Sílvia Maria Almeida de Souza, Gervásio Paulo da Silva, Giovani Brandão Mafra de Carvalho, José Ailton Conceição Bispo, Ernesto Acosta Martínez

**Affiliations:** ^1^Department of Technology, State University of Feira de Santana, Avenida Transnordestina, S/N^o^, 44.036-900, Feira de Santana-BA, Brazil; 2 School of Agronomy, Federal University of Goiás, Av. Esperança, s/n - Chácaras de Recreio Samambaia, 74.690-900, Goiânia – GO, Brazil; 3 Department of Education, Bahia State University, Rodovia Lomanto Jr, Br. 407 Km 127, s/n,48.970-000, Senhor do Bonfim – BA, Brazil

**Keywords:** rice bran extract, soybean meal extract, commercial supplement, honey must fermentation, mead production

## Abstract

**Research background:**

Due to the lack of nitrogen in honey, fermentation of honey must is limited or delayed, in addition to stimulating the production of unpleasant sensory compounds, such as sulfur derivatives. The use of natural supplements has been investigated as low-cost alternatives mainly to correct the nutritional deficiency of nitrogen in honey must in mead production.

**Experimental approach:**

Initially, the physicochemical characterization of the rice bran and soybean meal extracts was carried out. The fermentation of three yeasts (*Saccharomyces bayanus* Premier Blanc, *Saccharomyces cerevisiae* Montrachet and *Saccharomyces cerevisiae* Safbrew T-58) in honey must supplemented with 30 g/L rice bran or soybean meal extracts was evaluated. The trials were compared with the fermentations of the must with commercial supplement (30 g/L) and the control trials. Fermentations were carried out in Erlenmeyer flasks containing honey must with supplements, inoculated with 10^6^ cell/mL yeast and incubated at 30 °C for 264 h.

**Results and conclusions:**

There was significant difference in the evaluated properties of the extracts, with the exception of reducing sugars. The fermentations with soybean meal extract reached the highest cell concentrations, as well as the largest consumption of glucose, fructose and ethanol. The glycerol concentrations slightly increased when soybean meal extract and commercial supplement were used. The highest concentrations of succinic and acetic acids were registered in the control trials produced by *Saccharomyces* strains Premier Blanc, Montrachet and Safbrew T-58. Formic and lactic acids were not produced. Results showed that the extracts can be used as low-cost alternatives for correcting the nutritional deficiency of nitrogen in honey must since their effect was similar to that of synthetic supplement.

**Novelty and scientific contribution:**

The use of low-cost, unconventional supplements such as those used in this work, in addition to reducing the cost of the process by reducing fermentation time and providing nutrients needed to improve yeast metabolism, prevents the formation of undesirable compounds in the beverage due to prolonged fermentation time. It also makes it possible to add value to industrial by-products. Unconventional supplements have still been little tested in mead production.

## INTRODUCTION

Mead is a drink obtained from the alcoholic fermentation of diluted honey by yeast (*1*), whose production is not standardized, and therefore, winemaking techniques and ingredients are frequently used in its production (*2*). There are several studies that aim to optimize and consequently standardize the process of mead making using the selection of the type of honey (*3*), fermentation agent (*4*), cell concentration (*5*), process conditions (*6*) and supplements (*6*, *7*).

The use of supplements has been the focus of numerous research studies to speed up the fermentation of honey must. According to Mendes-Ferreira *et al*. (*8*), the fermentation may be limited or delayed mainly due to the lack of nitrogen in honey, which can additionally stimulate the production of unpleasant sensory compounds, such as sulfur derivatives.

Synthetic supplements have been more commonly used to correct the nutritional deficiency of must than natural ones. According to Sridee *et al*. (*9*), the use of low-cost nitrogen sources to replace commercial supplements, such as peptone and yeast extract has been continuously investigated; however, there are still few studies about their use in mead production. The interest in a better use of agroindustrial by-products has increased. Rice bran and soybean meal are examples of by-products widely used as supplements to obtain compounds of industrial interest (*10*). Rice bran contains 14-16% protein, and the protein nutritional value of this bran is relatively high due to an elevated concentration of lysine (*11*). It has good levels of vitamins and minerals, such as phosphorus and manganese (*12*). In industry, it is commonly used as a raw material for oil extraction, animal feed production, and less frequently, in the preparation of dietary products, multi-mix composition, and as a nitrogen source in the production of xylitol (*10*). Soybean meal is obtained from oil extraction and has approx. 48% protein, 35% carbohydrates, 10% water, 5% minerals and less than 1% fat (3 to 4% hydrolyzed fat) (*13*). There are many reports in literature that address the use of bran in animal feed, but there is also a high consumption of this bran as tofu in Asian countries (*14*).

Given the context, the present work aims to make use of unconventional supplements, mainly as nitrogen sources in honey must in the mead production. This work is unique, as it represents the first study in which agroindustrial waste was used in the preparation of supplements for mead production.

## MATERIALS AND METHODS

### Raw material

Floral honey [pH=3.4±0.0, total soluble solids (81.70±0.06) °Brix, composed of, in (*m*/*V*)/%: glucose (21.70±0.98), fructose (46.45±0.27), sucrose (7.70±0.01), in *w*/%: proteins (0.51±0.11), lipids (0.39±0.01), ash (0.07±0.01), and in *w*/(mg/kg): potassium (1062±3), sodium (584±4), phosphorus (168.57±0.82), calcium (50.96±0.65), magnesium (11.16±0.15), iron (2.07±0.16), manganese (1.13±0.08) and zinc (0.28±0.08)] (*4*) from Cooperativa of Ribeira do Pombal (COOOARP), Brazil, rice bran and soybean meal (local market, Feira de Santana, Brazil) were used.

### Yeast strains

The strain of *Saccharomyces bayanus* Premier Blanc (Red Star, Bastogne, Belgium) and two strains of *Saccharomyces cerevisiae*: Safbrew T-58 (Fermentis, Marcq-en-Barœul, France) and Montrachet (Red Star) were tested.

### Preparation of rice bran and soybean meal extracts

The solid (rice bran or soybean meal) and distilled water were added to glass bottles. Then, the mixtures (150 g/L) were autoclaved at 121 °C for 15 min and centrifuged at 2739×*g* for 10 min using Excelsa^®^ bench centrifuge (Fanem Ltda, São Paulo, Brazil) according to Araújo *et al*. (*4*).

### Preparation of the commercial supplement solution

The commercial supplement solution (150 g/L) was obtained by diluting in water the following reagents (Labsynth, São Paulo, Brazil; in g/L): yeast extract 36.8, malt extract 36.8, peptone 73.5, magnesium chloride 0.38, ammonium sulfate 2.25 and diammonium hydrogen phosphate 0.38 according to Amorim *et al*. (*7*). The solution was autoclaved at 121 °C for 15 min.

### Physicochemical characterization of rice bran and soybean meal extracts

The titratable acidity (%) was measured by volumetry with phenolphthalein indicator (Labsynth). The protein mass fraction (%) was determined by the Kjeldahl method, and ash (%) by calcination in an SSFM muffle furnace (SolidSteel, São Paulo, Brazil) (*15*).

The pH was determined with a digital pH meter (Instrutherm, São Paulo, Brazil), and total soluble solids (°Brix) by refractometry, using an AR200 digital device (Reichert, São Paulo, Brazil) at 20 °C.

Carbohydrate mass fraction (%) was determined according to the method proposed by Trevelyan and Harrison (*16*), and reducing sugars (%) were quantified according to Miller (*17*) by spectrophotometry (UV mini-1240; Shimadzu, São Paulo, Brazil) at a wavelength of 540 nm, using dinitrosalicylic acid (DNS) as a reagent.

The concentration of assimilable nitrogen (mg/L) was determined using the titrimetric method with standardized 0.1 M NaOH solution according to Zoecklein *et al*. (*18*). The mass fraction of elements (mg/100 g) Na and K was quantified by atomic emission and Ca, Mg, Zn, Fe and Mn by atomic absorption, while P was analyzed using the ammonium trioxovanadate(V) and 482 UV-Vis spectrophotometry (Femto, São Paulo, Brazil) (*19*).

### Inoculum preparation

The yeasts were weighed according to the manufacturer's instructions and transferred to 125-mL Erlenmeyer flasks containing 50 mL of honey must (30 °Brix). The mixtures were shaken (Tecnal TE-420, São Paulo, Brazil) at 150 rpm and 30 °C for 24-48 h to reach 10^7^ cell/mL.

### Fermentation tests

The honey was diluted in sterile water in 500-mL Erlenmeyer flasks. Soybean meal, rice bran extracts or commercial supplements were added to the mixture of honey with distilled water. The pH of the mixtures was adjusted to 5.0 using calcium carbonate.

The supplemented must was inoculated according to Amorim *et al*. (*7*) and Araújo *et al*. (*4*). Subsequently, the Erlenmeyer flasks were sealed with a stopper coupled with an airlock valve containing 70% ethyl alcohol and placed in a TE-371 biochemical oxygen demand (BOD) oven (Tecnal) at 30 °C for 264 h.

The experiments were carried out in triplicate and, for each yeast strain, the addition of soybean meal and rice bran extracts (30 g/L) was evaluated and compared to the must fermentation with commercial supplement (30 g/L) and the controls.

### Analytical monitoring of the fermentation

#### Cell concentration

The fermentation was monitored every 24 h to determine the cell number by counting in a Neubauer chamber, and the number of viable and non-viable cells (*20*).

#### Concentrations of sugars, ethanol and organic acids

The concentrations of glucose, fructose, sucrose, ethanol, glycerol and organic acids (acetic, lactic, succinic and formic) during fermentation were quantified by high performance liquid chromatography (ultimate 3000; Dionex, Sunnyvale, CA, USA) using a Rezex ROA H+ column (300 mm×7.8 mm; Phenomenex, Torrance, CA, USA) and Shodex RI-101 detector (Showa Denko, Tokyo, Japan) for sugars and alcohol, using 0.005 M H2SO4 as eluent, at a flow rate of 0.6 mL/min and column temperature of 45 °C.

### Statistical analysis

The analysis of variance (ANOVA) and Tukey's test served to identify significant differences between the mean values of the obtained results using the SISVAR program v. 5.6 (*21*). Differences between the mean values at the 5% level (p<0.05) were considered significant.

## RESULTS AND DISCUSSION

### Physicochemical properties of rice bran and soybean meal extracts

As shown in Table 1, there was a significant difference (p≤0.05) in all parameters evaluated for the rice bran and soybean meal extracts, with the exception of reducing sugars.

**Table 1:** Physicochemical characteristics of rice bran and soybean meal extracts

Parameter	Rice bran extract	Soybean meal extract
pH	(6.10±0.00)^b^	(6.50±0.00)^a^
*w*(TTA)/%	(2.2±0.1)^b^	(1.4±0.25^a^
TTS/^o^Brix	(1.60±0.06)^b^	(6.70±0.00)^a^
*w*(reducing sugar)/%	(0.11±0.00)^a^	(0.12±0.00)^a^
*w*(total carbohydrate)/%	(1.60±0.03)^b^	(6.52±0.05)^a^
*w*(protein)/%	(0.42±0.03)^b^	(1.70±0.07)^a^
*γ*(nitrogen)_assimilable_/(mg/L)	(16.9±1.6)^b^	(69.10±0.06)^a^
*w*(ash)/%	(0.23±0.01)^b^	(0.72 ±0.01)^a^
*w*(mineral)/(mg/100 g)		
Calcium	(2.83 ±0.00)^b^	(11.0±0.2)^b^
Magnesium	(14.78±0.09)^b^	(27.2 ±0.2)^a^
Zinc	(0.14±0.03)^b^	(0.30 ±0.01)^a^
Iron	(0.88±0.02)^b^	(2.40±0.01)^a^
Sodium	(5.40±0.03)^b^	(7.5±0.1)^a^
Potassium	(137.7±2.2)^b^	(506±10)^a^
Phosphor	(2.2±0.2)^b^	(18.5±0.7)^a^
Manganese	n.d.	n.d.

n.d.=not detected, TTA=total titratable acidity, TTS=total soluble solids

Rice bran extract had a slightly lower pH (6.1) than soybean meal extract (6.5), and consequently, 1.55 times higher total titratable acidity (2.21%) (Table 1). Feddern *et al*. (*22*) determined the acidity and pH values (2.1% and 6.5 respectively) of rice bran. Ginger-Reverdin *et al*. (*23*) found a close pH value (6.76) and lower total titratable acidity (0.82%) of soybean meal. Acidity and pH are the parameters that indicate the conservation state of the raw material. The acidity may increase during storage, which may assist the fermentation, as well as lipid hydrolysis (*24*).

The content of total soluble solids and carbohydrates in soybean meal extract was ​​approx. four times higher (6.70 °Brix and 6.52% respectively) than in rice bran extract (Table 1). Carbohydrate mass fractions ​​between 25.71 and 32.89% were verified by Garcia *et al*. (*25*) for rice bran. According to Malekian *et al*. (*11*), the carbohydrates present in the rice bran are hemicellulose (8.7–11.4%), cellulose (9.0–12.8%), starch (5–15%) and β-glucans (1%). According to Choct *et al*. (*26*), soybean meal has around 35% carbohydrates, composed mainly of sucrose, stachyose, raffinose, pectin, cellulose and starch.

The mass fractions of reducing sugars were similar (0.11 and 0.12%) in both extracts (Table 1). Garcia *et al*. (*25*) analyzed rice bran that showed higher mass fractions of reducing sugars (1.23–2.60%). Approx. 0.6% reducing sugars in conventional soybean meal was reported by Paris (*27*).

The rice bran and soybean meal extracts contained respectively 0.42 and 1.70% protein and 16.9 and 69.10 mg/L assimilable nitrogen (Table 1). Bhosale and Vijayalakshmi (*28*) reported 17.5% protein in stabilized rice bran and 19.25% in probiotic rice bran. In soybean meal 48.38% protein was found by Gerber *et al*. (*29*). Regarding the assimilable nitrogen content, the value obtained for soybean meal was higher than the one reported by Araújo *et al*. (*4*) for cowpea extract (45.73%) used in the mead fermentation. Assimilable nitrogen is a fundamental component in fermentation of beverages, such as wine and mead. According to Iglesias *et al*. (*30*), an inadequate amount of assimilable nitrogen in fermentation can harm yeast growth, extend the fermentation and decrease ethanol productivity.

The ash content was approx. three times higher in the soybean meal extract (0.23%) than in rice bran extract (Table 1). Sairam *et al*. (*31*) reported a value of 7.4% ash in rice bran. Ghadge *et al*. (*32*) found 6.89% ash in soybean meal. According to Vicentini-Polette *et al*. (*33*), the ash content is linked to the minerals present in the product.

The highest mineral mass fractions (mg/100 g) were found in the soybean meal extract: calcium (11.0), magnesium (27.2), zinc (0.30), iron (2.40), sodium (7.5), potassium (506) and phosphorus (18.5), which were 3.87, 1.84, 2.14, 2.72, 1.39, 3.67 and 8.30 times higher, respectively, than in the rice bran extract (Table 1). It was not possible to detect the presence of manganese in both extracts. These minerals positively influence cell growth and fermentation (*34*). Carvalho *et al*. (*35*) also found higher concentrations of minerals in the soybean extract. The authors reported that soybean and brown rice extracts, respectively, had the following mineral mass fractions (mg/kg): calcium (15.75 and 12.03), magnesium (28.50 and 1.69), zinc (1.82 and 0.18), iron (4.31 and 0.77) and manganese (0.16 and 0.15).

### Influence of rice bran and soybean meal extracts on cell growth of the selected yeast strains

**Fig. 1:**
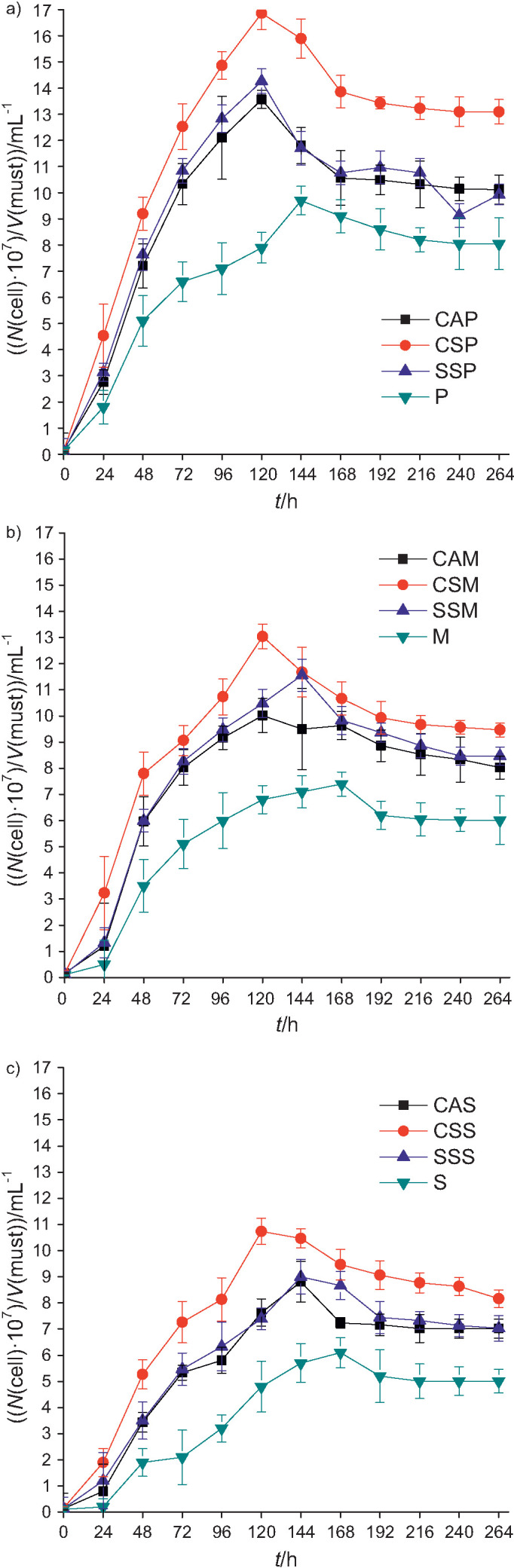
Cell concentration during fermentation of honey must supplemented with 30 g/L of soybean meal extract (circles), bran extract (squares), commercial supplement (triangle) and without supplement (down pointing triangle) using commercial yeast: a) *Saccharomyces bayanus* Premier Blanc, b) S*accharomyces cerevisiae* Montrachet and c) S*accharomyces cerevisiae* Safbrew T-58

Fig. 1 shows the cell growth profile of the yeast strains Premier Blanc, Montrachet and Safbrew T-58 during the fermentation of honey must supplemented with 30 g/L rice bran extract, soybean meal extract or commercial supplement, and in the controls.

The evaluated supplements promoted the cell growth of all yeasts when compared to the control assays; however, the highest cell concentrations ​​were obtained in fermentations with soybean meal extract after 264 h of fermentation regardless of the yeast strain. Thus, it can be inferred that soybean meal extract provided necessary nutrients in an ideal concentration for cell growth, such as assimilable nitrogen and minerals, since it had higher concentrations of these components than rice bran extract (Table 1). The lack or limitation of these nutrients and other growth factors compromise the development of yeast (*36*). Schwarz *et al*. (*37*) evaluated the influence of honey must supplementation with nitrogen, minerals and vitamins on the mead production and found that these components favored the production of yeast biomass.

The effect of supplementation can be clearly seen on the growth of all yeasts throughout the fermentation compared to the controls (Fig. 1). Almeida *et al*. (*2*) evaluated the effect of commercial nitrogen sources (diammonium phosphate and ammonium sulphate) on the fermentation of honey must (25 °Brix) inoculated with 10^6^ cell/mL of *Saccharomyces cerevisiae* JP14 at 25 °C for 28 days. These authors reported that up to 120 h of fermentation, both supplements helped increase the concentration of viable cells.

Comparison of the cell growth profiles of the three yeasts in the media supplemented with soybean meal extract, rice bran extract or the assays with commercial supplement shows that the cell concentrations obtained during fermentation were similar in the media with rice bran extract and commercial supplement and lower than those obtained in trials with soybean meal extract (Fig. 1).

Using soybean meal extract, *S. bayanus* Premier Blanc reached higher maximum cell concentration (16.9·10^7^ cell/mL), followed by *S. cerevisiae* Montrachet (13.0·10^7^ cell/mL) and *S. cerevisiae* Safbrew T-58 (10.7·10^7^ cell/mL) after 120 h of fermentation. On the other hand, in the fermentation of must with rice bran extract, Premier Blanc strain reached 13.6·10^7^ cell/mL and Montrachet 10.0·10^7^ cell/mL after 120 h, and Safbrew T-58 reached 8.8·10^7^ cell/mL after 144 h (Fig. 1).

The maximum values of cellular of the commercial supplement media were slightly higher than those obtained in the trials with rice bran extract. Strains Premier Blanc, Montrachet and Safbrew T-58 reached the following maximum cell concentrations (in cell/mL): 14.3·10^7^ in 120 h, 11.6·10^7^ after 144 h and 9.0·10^7^ after 144 h, respectively. In the control trials, the values of the maximum cell concentrations were lower than in the other trials (9.7·10^7^ cell/mL after 144 h, 7.4·10^7^ cell/mL and 6.1·10^7^ cell/mL after 168 h) obtained by Premier Blanc, Montrachet and Safbrew T-58 strains, respectively (Fig. 1).

Amorim *et al*. (*7*) evaluated the influence of the addition of acerola pulp (10, 15, 20, 25 and 30%) in the fermentation of honey must (30 °Brix) by *Saccharomyces cerevisiae* AWRI796 (10^7^ cell/mL) at 30 °C and pH=5.0. The increasing mass fractions of pulp progressively supported cell growth, which reached maximum concentration (2.09·10^8^ cell/mL) after 288 h of fermentation. Araújo *et al*. (*4*) supplemented the honey must (30 °Brix) with cowpea extract (0, 5 and 30 g/L); the medium was inoculated with 10^6^ cell/mL and fermentations were conducted at 30 °C and pH=5.0 for 264 h. According to these authors, cell growth was supported by higher concentration of cowpea extract (30 g/L), with higher cell concentration (19.0·10^7^ cell/mL) of *S. bayanus* Premier Cuvée after 168 h, 11.3·10^7^ cell/mL of *S. bayanus* Premier Blanc after 120 h and 11.1·10^7^ cell/mL of *S. cerevisiae* Safbrew T-58 after 96 h. Pereira *et al*. (*36*) evaluated the cell growth profile of two strains of *S. cerevisiae* (QA23 and ICV D47) in honey must (37 *m*/*V*), supplemented with salts, vitamins and the combination of both nutrients, which were inoculated with 10^5^ cell/mL and fermented at 25 °C for 288 h. These authors found that the growth profile was influenced more by the yeast strain than by the supplements added to the must. The QA23 strain reached a maximum concentration of approx. 10^8^ cell/mL, whereas the ICV D47 strain reached a concentration of 7 to 8·10^7^ cell/mL after 48 h of fermentation.

### Influence of rice bran and soybean meal extracts on sugar profile and ethanol concentrations during fermentation of honey must using commercial yeasts

**Fig. 2:**
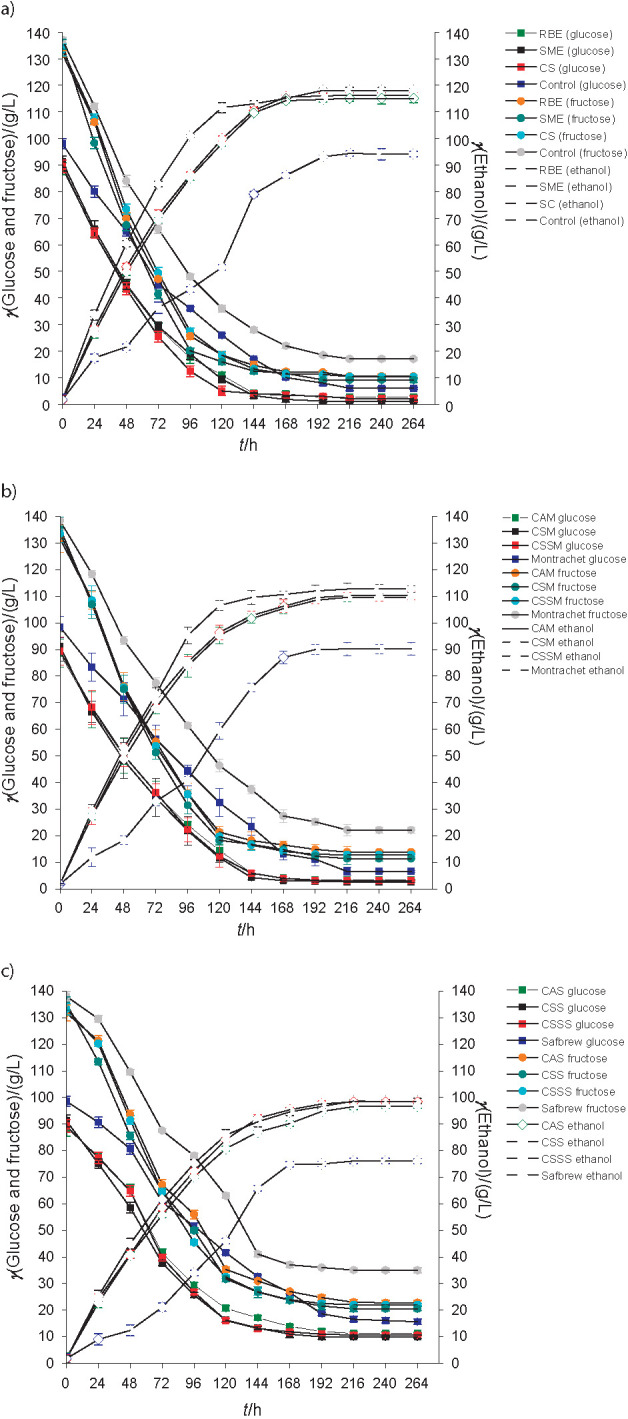
Profile of sugars (glucose and fructose) and ethanol concentrations during the fermentation of honey must supplemented with 30 g/L of rice bran (RBE) and soybean meal extracts (SME), commercial supplement (CS) and without supplements using commercial yeast: a) *Saccharomyces bayanus* Premier Blanc, b) S*accharomyces cerevisiae* Montrachet and c) S*accharomyces cerevisiae* Safbrew T-58

Fig. 2 shows that the consumption of glucose and fructose by strains Premier Blanc, Montrachet and Safbrew T-58 occurred simultaneously both in the fermentation of supplemented musts and in the controls. Similar behavior was observed by Araújo *et al*. (*4*), where in all trials performed (supplemented with cowpea extract and control), both sugars were consumed at the same time.

In general, it is observable that regardless of the yeast, in the trials with supplements the consumption of sugars and production of ethanol were higher than in the controls (Fig. 2), and it can be inferred that the rice bran and soybean meal extracts provided essential nutrients to the media in adequate concentrations for better yeast fermentation performance. According to Gibson (*38*) and Silva *et al*. (*39*), honey has a low amount of nitrogen, minerals and vitamins, so the correction of the nutritional deficiencies of the must can reduce the sensitivity of the yeast to stress, improving the fermentative performance.

There was no significant difference (p≤0.05) in sugar consumption in fermentations of supplemented musts. However, the consumption of glucose (98.6, 97.1 and 89.1%) and fructose (93.2, 91.6 and 84.8%) by strains Premier Blanc, Montrachet and Safbrew T-58 was higher when using soybean meal extract than rice bran extract (97.1, 96.0 and 87.4% glucose) and (92.0, 89.5 and 82.7% fructose) (Table 2).

**Table 2:** Consumption of glucose and fructose and final concentration of ethanol (g/L) in the fermentation of honey must with 30 g/L of rice bran (RBE), soybean meal extracts (SME), commercial supplement (CS) and without supplements using *Saccharomyces bayanus* Premier Blanc, *Saccharomyces cerevisiae* Montrachet and S*accharomyces cerevisiae* Safbrew T-58

Yeast strain	Supplement	*w*(glucose)_consumed_/%	*w*(fructose)_consumed_/%	*γ*(ethanol)/(g/L)
Premier Blanc	SME	(98.6±1.2)^a^	(93.2±1.1)^a^	(118.1±2.7)^a^
	RBE	(97.1±2.0)^a^	(92.0±1.2)^a^	(115.0±2.2)^a^
	CS	(97.7±2.1)^a^	(92.3±1.0)^a^	(116.3±2.0)^a^
	Control	(91.9±0.6)^b^	(87.5±0.6)^c^	(94.1±2.2)^c^
				
Montrachet	SME	(97.1±0.8)^a^	(91.6±1.3)^a^	(112.7±4.8)^a^
	RBE	(96.0±1.8)^a^	(89.5±0.9)^a^	(109.0±2.3)^a^
	CS	(96.5±1.8)^a^	(90.4±1.2)^a^	(110.3±3.6)^a^
	Control	(90.3±0.5)^b^	(84.0±1.56)^b^	(90.3±1.3)^b^
				
Safbrew T-58	SME	(89.1±0.9)^a^	(84.8 ±1.0)^a^	(98.6±2.0)^a^
	RBE	(87.4±1.0)^a^	(82.7±1.2)^a^	(96.8±2.0)^a^
	CS	(88.0 ±1.9)^a^	(83.5±2.1)^a^	(98.40±1.0)^a^
	Control	(83.6±1.0)^b^	(74.60±1.07)^b^	(76.1±1.0)^b^

On the other hand, with the use of the commercial supplement, the consumption of glucose and fructose was closer to that obtained in fermentation media with rice bran extract (Table 2). In the controls, the values of consumption of these sugars differed significantly (p≤0.05) compared to all supplemented media, having lower values ​​of the consumption of glucose (91.9, 90.3 and 83.6%) and fructose (87.5, 84.0 and 74.6%) by Premier Blanc, Montrachet and Safbrew T-58, respectively (Table 2).

Araújo *et al*. (*4*) reported higher values of glucose (99.8 and 99.8%) and fructose (95.5 and 85.6%) consumption, respectively, when they evaluated the performance of strains Premier Blanc and Safbrew T-58 in the fermentation of honey must supplemented with 30 g/L cowpea extract. Kawa-Rygielska *et al*. (*40*) used fruits and herbs as supplements in the fermentation of honey must (34 °Brix) by *S. bayanus* Safspirit Fruit at 22 °C for 16 days. These authors reported higher glucose consumption (77%) in the trials with grape seeds and lower (60%) in the controls, while fructose consumption was on average 45% in all trials.

Lower concentrations of sugars are observed in mead obtained from must supplemented with soybean meal extract (glucose 1.2, 2.6 and 10.0 g/L, and fructose 9.2, 11.4 and 20.51 g/L), followed by the concentrations obtained from the trials with rice bran extract (glucose 2.6, 3.5 and 11.2 g/L, and fructose 10.5, 13.9 and 22.7 g/L) as shown in Fig. 2. Similar values for glucose (2.1, 3.1 and 10.7 g/L) and fructose (10.3, 12.8 and 22.1 g/L) were obtained in the mead produced from the must containing commercial supplements.

On the other hand, as expected, in the control trials, the final concentrations of these sugars in the mead were higher (glucose 6.0, 6.6 and 10.7 g/L, and fructose 17.1, 22.1 and 35.0 g/L). Gomes *et al*. (*41*) reported lower concentrations of glucose (2.55-5.11 g/L) and fructose (1.51 and 27.6 g/L) in the mead obtained by the fermentations of honey must (395 g/L) at 20 to 30 °C for 15 days. Lower concentrations of sugars are indicative of complete fermentation avoiding re-fermentation, which can cause contamination by undesirable microorganisms and consequently the production of odors not characteristic of the product (*41*). Silva *et al*. (*42*) produced dry and sweet mead by the fermentation of honey must (28.2 °Brix) at 25 °C for 13 days and reported glucose concentrations of 4.9 and 50.5 g/L, and fructose concentrations of 5.45 and 99.9 g/L in dry and sweet mead, respectively.

The obtained mead can be characterized as smooth, since the residual sugar concentration was higher than 3 g/L, as recommended by the Brazilian legislation (*43*).

In all trials where the medium was supplemented, ethanol production (31.6-110.5 g/L; 4-14%) was observable (Fig. 2) from 24 to 48 h of fermentation, which is characteristic alcoholic content of mead according to the Brazilian legislation (*43*). However, in the control trials, mead containing this volume fraction of ethanol was solely obtained after 72 to 96 h. Hernández *et al*. (*44*) reported between 2 and 8% ethanol from the fermentation of honey must samples (24 °Brix), separately supplemented with yeast extract, pollen, pretreated pollen and the mixture of pollen and ammonium dihydrogen phosphate, fermented by strains of *S. cerevisiae* (UVAFERM BC, FERBLANC AROM and LALVIN QA23) for 24–36 h.

Difference in the ethanol content among the mead samples fermented with supplements was not significant, except in the controls, where they were lower (Table 2).

With the use of soybean meal extract, ethanol concentrations were slightly higher (118.0 g/L (*φ*=15%), 112.7 g/L (*φ*=14.3%), 116.3 g/L (*φ*=14.7%) and 98.6 g/L (*φ*=12.5%)) than those obtained in fermentation with rice bran extract (115.0 g/L (*φ*=14.6%), 109.7 g/L (*φ*=13.9%) and 96.8 g/L (*φ*=12.3%)) by strains Premier Blanc, Montrachet and Safbrew T-58, respectively. In the fermentation using the must supplemented with commercial supplement, the ethanol concentrations of the mead were close to those obtained with the use of soybean meal and rice bran extracts (Fig. 2). Lower volume fractions of ethanol (10.7–11.4%) in the must supplemented with diammonium phosphate and 10.8% in the control trials were obtained by Mendes-Ferreira *et al*. (*45*) in the fermentation of honey must (37 g/100 mL) by *S. cerevisiae* UCD522 at 22 °C for 25 days. Amorim *et al*. (*7*) reported that at the optimum point in the fermentation of honey must supplemented with acerola, 120.1 g/L (15.2%) of ethanol was obtained.

### Influence of rice bran and soybean meal extracts on the production of glycerol and organic acids by the three strains

**Fig. 3:**
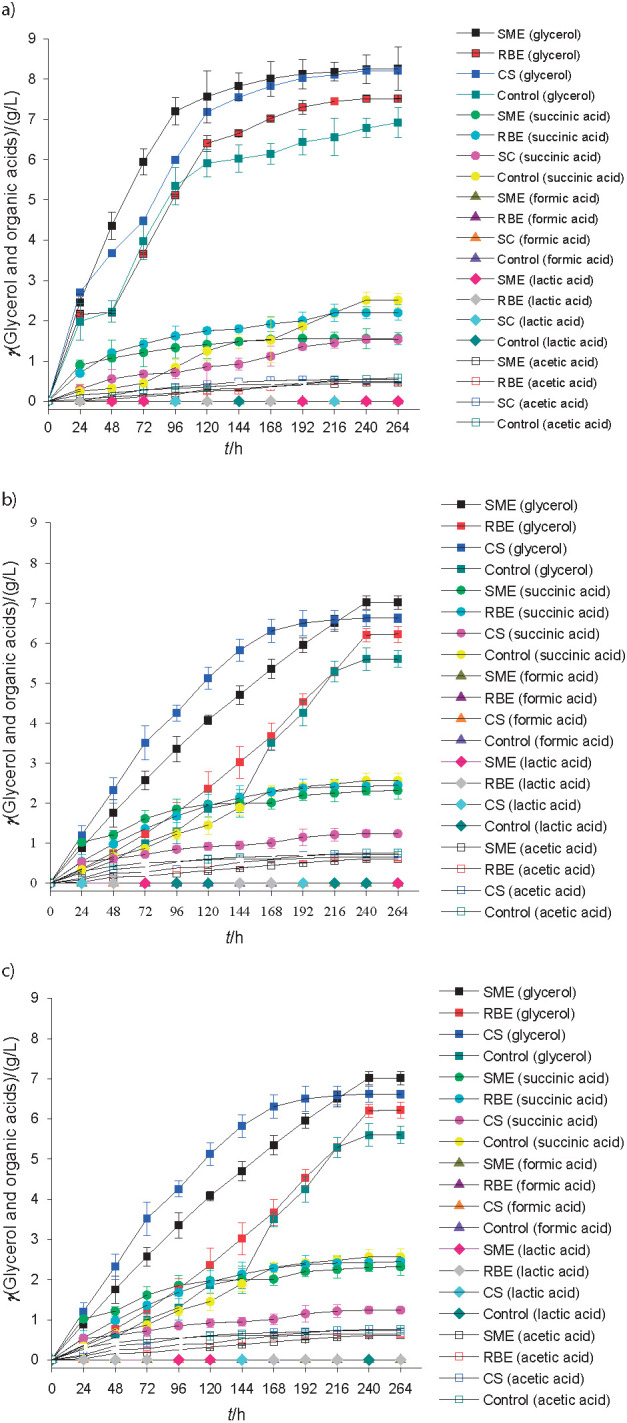
Profile of glycerol and organic acids (acetic, formic, lactic, acetic and succinic) during the fermentation of honey must supplemented with 30 g/L rice bran (RBE), soybean meal extracts (SME), commercial supplement (CS) and without supplements using commercial yeast: a) *Saccharomyces bayanus* Premier Blanc, b) S*accharomyces cerevisiae* Montrachet and c) *Saccharomyces cerevisiae* Safbrew T-58

The glycerol production profile depended on the used yeast (Fig. 3). Strain Premier Blanc produced mead with higher and similar concentrations of glycerol (8.3, 8.2 and 7.5 g/L) in the fermentation of the must supplemented with soybean meal extract and commercial supplement than the trials with rice bran extract, respectively.

Araújo *et al*. (*4*) reported that the highest concentrations of glycerol (10.08 and 8.49 g/L) obtained by Premium Blanc and Safbrew T-58 strains were observed in trials with a higher concentration of supplement (30 g/L). Similar to the results obtained in this study, they reported that lower concentrations of this by-product were observed in the control trials. Gomes *et al*. (*41*) reported glycerol concentrations between 5.42 and 6.89 g/L in the mead obtained by the fermentation of honey must supplemented with different concentrations of nutrient salts at different temperatures.

Glycerol is a by-product of fermentation that contributes to sensory characteristics, generally found in the range of 7 to 10% of the produced ethanol (*46*). According to Adamenko *et al*. (*47*), the glycerol amount can be affected by the presence of organic acids, such as succinic, formic and acetic. Organic acids have an influence on the sensory characteristics and stability of alcoholic beverages (*48*). Formic and lactic acids were not detected in the trials (Fig. 3).

In fermentations of honey must supplemented with soybean meal extract, the concentration of succinic (1.56, 2.04 and 2.32 g/L) and acetic (0.47, 0.52 and 0.61 g/L) acids in the mead were slightly lower than the concentrations observed in the trials with rice bran extract: succinic (2.20, 2.40 and 2.45 g/L) and acetic (0.50, 0.60 and 0.65 g/L) acids after fermentation by Premium Blanc, Montrachet and Safbrew T-58 strains, respectively. On the other hand, with the use of commercial supplement, the yeasts produced lower concentrations ​​of succinic acid than those obtained in the trials with soybean meal and rice bran extracts, and higher concentrations of acetic acid (Fig. 3). In trials without the addition of supplements, higher concentrations ​​of succinic (2.51, 2.83 and 2.56 g/L) and acetic (0.58, 0.72 and 0.76 g/L) acids were produced by Premium Blanc, Montrachet and Safbrew T-58, respectively, than in trials in which the must was supplemented. The values ​​found for succinic acid are in the range (0.34 and 3.98 g/L) reported by Švecová *et al*. (*49*), but some values ​​are below the range 0.62-16.61 g/L for acetic acid. Lower concentrations (0.2 and 1.0 g/L) of succinic and acetic acids, respectively, were reported by Sroka and Satora (*50*) in the mead obtained from honey must (1:3).

## CONCLUSIONS

Rice bran and soybean extracts obtained from low-cost raw materials provided the honey mead with the nutrients needed for better yeast performance as efficiently as commercial supplement, thus reducing the time of the fermentation and consequently the cost of the final product. *Saccharomyces bayanus* Premier Blanc and *S. cerevisiae* Montrachet yeasts performed better than Safbrew T-58, regardless of the used extract. It was concluded that the tested extracts have the potential to be used as innovative supplements in the mead production, and furthermore, to add value to industrial by-products.
